# A systematic review and meta-analysis of Gustafson’s six dental variables for age estimation in adults

**DOI:** 10.1038/s41598-026-62961-x

**Published:** 2026-07-21

**Authors:** Rizky Merdietio Boedi, Nikolaos Angelakopoulos, Gabriel M. Fonseca, Galina Zolotenkova, Ademir Franco

**Affiliations:** 1 Division of Forensic Odontology and Medicolegal, Faculty of Medicine, Universitas Diponegoro, Semarang, Indonesia; 2 Department of Forensic Medicine, Sechenov First Moscow State Medical University, Moscow, Russian Federation; 3 Department of Orthodontics and Dentofacial Orthopedics, University of Bern, Freiburgstrasse 7, Bern, 3010 Switzerland; 4 Division of Anatomy and Forensic Dentistry, Faculdade São Leopoldo Mandic, São Paulo, Brazil; 5 Faculty of Dentistry, Centro de Investigación en Odontología Legal y Forense (CIO), Universidad de La Frontera, Temuco, Chile

**Keywords:** Dental age assessment, Forensic dentistry, Gustafson, Meta-analysis, Computational biology and bioinformatics, Diseases, Health care, Medical research

## Abstract

Dental age estimation (DAE) by means of direct visualisation remains a fundamental tool in forensic odontology, particularly for Disaster Victim Identification (DVI) in resource-limited settings. This systematic review and meta-analysis aimed to calculate the pooled correlation coefficient between chronological age (CA) and six Gustafson-derived dental variables in modern adults. Following PRISMA guidelines, five databases were searched until June 2025, resulting in 27 included studies, with 19 utilized in a three-level random-effects meta-analysis. The overall weighted correlation between CA and Gustafson-derived variables was moderate (*r* = 0.67, $\:{I}_{b}^{2}$ = 0.93), though significant heterogeneity was observed with low certainty due to method variability. Subgroup analyses indicated that the six variables do not contribute equally to age prediction; transparency of the root gives the highest correlation (*r* = 0.75), while root resorption showed the lowest correlation (*r =* 0.28). Additionally, sectioned teeth showed a higher correlation than intact teeth, although this difference was not statistically significant. In conclusion, while direct visualisation of Gustafson-derived variables shows utility for DAE, high between-study heterogeneity and low certainty of evidence dictate cautious application and interpretation of the current results. Furthermore, although tooth sectioning may improve correlation, using destructive methods requires balancing potential analytical benefits against ethical and legal constraints.

**Supplementary Information:**

The online version contains supplementary material available at 10.1038/s41598-026-62961-x.

## Introduction

Gustafson (1950) established a foundational method for dental age estimation (DAE) in adults based on six age-related regressive changes in dental tissues: attrition, periodontosis, secondary dentin deposition, cementum apposition, root resorption, and transparency of the root^1^. While these six variables remain central to adult DAE, the primary challenge in the adult population remains achieving an accurate estimation of chronological age (CA). This is mainly due to the process of observing age-related regressive changes, which are highly susceptible to confounding external factors. For instance, attrition can be accelerated by malocclusion, such as a deep bite^2^. Secondary dentin formation cannot be distinguished from tertiary dentin using macroscopic observation or radiographic techniques^3^. Additionally, since tertiary dentin can be stimulated by dental trauma^4^, it introduces variability that is independent of CA.

To address these challenges and improve the reliability of the models, researchers have developed advanced methodological approaches. These methods range from detailed imaging techniques, such as volumetric assessment using Cone-Beam Computed Tomography^5^, to molecular methods that bypass morphological analysis entirely, such as DNA methylation and aspartic acid racemisation^6^. Although these technologies have demonstrably enhanced the reliability of adult DAE, the fundamental practice of direct visualisation DAE continues to be widely used^7^.

The popularity of direct visualisation DAE is primarily driven by its practical and cost-effective applications in various scenarios. It is indispensable in resource-limited settings where advanced imaging or laboratory facilities are unavailable, and it provides a means for rapid preliminary age assessment in scenarios such as mass disaster victim identification (DVI)^8, 9^. Over the decades, methods for the evaluation of Gustafson’s six variables have undergone numerous iterations, including modifications to scoring criteria and statistical formulae; however, the core principles remain. Given the long history and varied applications of these techniques, a comprehensive synthesis of their effectiveness is necessary.

Therefore, to clarify the significance of these foundational regressive changes when assessed under direct visualisation, this systematic review and meta-analysis is guided by the following research question: “In adults, what is the pooled correlation coefficient between CA and each of the six Gustafson-derived dental variables when assessed through direct visualisation of modern human teeth?”

## Materials and methods

### Eligibility criteria

This review was performed following the Preferred Reporting Items for Systematic Reviews and Meta-Analyses (PRISMA) guidelines^10^. The research question stated previously was established based on population (P), exposure (E), comparison (C), and outcome (O) with the following criteria: P = modern human populations, E = Measurement or Scoring of Individual Gustafson’s derived variables via direct visualisation of teeth, C = CA, and O = the correlation coefficient between the variables and CA. The six Gustafson-derived variables were determined as attrition, periodontosis, secondary dentin deposition, cementum apposition, root resorption, and transparency of the root^1^. This study was registered with PROSPERO under the registration number CRD420251157113 (https://www.crd.york.ac.uk/PROSPERO/view/CRD420251157113).

### Information sources

An electronic search (24 June 2025) was conducted in five databases: PubMed, Scopus, Virtual Health Library, SciELO and Open Access Theses and Dissertation (OATD). The latter representing the grey literature to reduce publication bias.

### Search strategy

Search strings were established based on their capability to capture the context of the morphological direct visualisation of teeth, together with search strings related to each of the six variables and methodology derivation of Gustafson, such as, but not limited to, Lamendin^11^, Johanson^12^, or Dalitz methods^13^. These initial search strings were then modified to align with Medical Subject Headings (MeSH) and Descriptors in Health Sciences (DeCS), using a combination with Boolean operators AND/OR (Table 1).

**Table 1:** Search Strategy for Each Database. The Boolean Operators used were AND, OR, or Asterisks.

Database	Search terms
PubMed	(“dental age estimation” OR “Age Determination by Teeth“[Mesh]) AND (“adult*” OR “morpholog*” OR “extrac*” OR “Tooth Attrition“[Mesh] OR “recession” OR “Gingival Recession“[Mesh] OR “Dental Cementum“[Mesh] OR “translucen*” OR “Dentin, Secondary“[Mesh] OR “denti*” OR “gustafson” OR “lamendin” OR “johanson” OR “dalitz”)
Scopus	TITLE-ABS-KEY ( ( “Dental Age Estimation” OR “Age Determination by Teeth” ) AND ( “adult” OR “Morpholog*” OR “extrac*” OR “attrition” OR “recession” OR “cementum” OR “Dental Cementum” OR “Translucen*” OR “secondary denti*” OR “Gustafson” OR “Lamendin” OR “Johanson” OR “Dalitz” ) ) AND PUBYEAR > 1989 AND ( LIMIT-TO ( SRCTYPE, “j” ) ) AND ( LIMIT-TO ( DOCTYPE, “ar” ) )
SciElo	(“Age Estimation” OR “Age Determination by Teeth” OR “dental age estimation” ) AND ( “adult” OR “Morpholog*” OR “extrac*” OR “attrition” OR “recession” OR “cementum” OR “Dental Cementum” OR “Translucen*” OR “secondary denti*” OR “Gustafson” OR “Lamendin” OR “Johanson” OR “Dalitz”)
Virtual Health Library	( “Age Determination by Teeth” OR “dental age estimation” ) AND ( “adult” OR “Morpholog*” OR “extrac*” OR “attrition” OR “recession” OR “cementum” OR “Dental Cementum” OR “Translucen*” OR “secondary denti*” OR “Gustafson” OR “Lamendin” OR “Johanson” OR “Dalitz”)
OATD	( “Age Determination by Teeth” OR “dental age estimation” ) AND ( “adult” OR “Morpholog*” OR “extrac*” OR “attrition” OR “recession” OR “cementum” OR “Dental Cementum” OR “Translucen*” OR “secondary denti*” OR “Gustafson” OR “Lamendin” OR “Johanson” OR “Dalitz”)

### Selection process

This study included full-text peer-reviewed articles, primarily observational or cross-sectional studies, that reported the correlation coefficient of Gustafson variables or their derivations for intact or sectioned teeth in modern humans with known chronological age. Where the correlation coefficient was not reported but the coefficient of determination (R²) from a single-predictor regression of CA on the Gustafson variable was provided, the correlation was reconstructed as r = √R²; the sign of r was assigned according to the direction of association explicitly reported in the original study (positive for an increasing relationship with age, negative otherwise). Only single-predictor R² values were used; multivariable R² values, which combine the contributions of multiple predictors and cannot be unambiguously back-converted, were excluded.

The studies included were published after 1990. This restriction was applied because of the scarcity of metadata and the limited availability of online journal copies. Studies employing the same methodology in archaeological research or reporting correlation values derived from multivariate analyses were excluded. Systematic reviews, abstracts, books, book chapters, editorials, and letters to the editor were excluded in the analysis.

Study selection was performed independently by two authors (RMB and NA), and any disagreement for each stage (i.e., title, abstract, full text) was resolved by consensus. When consensus was not reached, a third author (AF) adjudicated. Metadata were managed using EndNote 20 (Thomson Reuters, Toronto, Canada). The initial selection involved automatic duplicate detection using the built-in function of EndNote 20. Manual duplicate detection was performed using Microsoft Excel 365 (Microsoft Ltd., Washington, USA). The second and third selection phases involved screening the study titles and abstracts accordingly. In cases of uncertainty, the articles were advanced to the next selection stage. Finally, full texts were assessed for eligibility, and studies that met the eligibility criteria were subjected to data extraction. Data extraction was done by one author (RMB).

### Data collection

The extracted data included authors’ names, year of publication, study population, sample size, age range, teeth examined, methodology, each of the Gustafson’s variables, and correlation coefficient between these variables and CA.

### Study risk of bias assessment

The risk of bias was qualitatively assessed using the Joanna Briggs Institute Critical Appraisal Tools for cross-sectional studies^14^. Each study was independently evaluated by two observers (RMB and NA) using a critical appraisal checklist. The initial agreement for the appraisal, measured using Cohen’s kappa, was 0.81. Discrepancies were resolved through discussions. Positive responses were categorized as follows: under 50% for high risk of bias, 50–69% for moderate risk, and 70% or above for low risk of bias.

Small-study effects and potential publication bias were evaluated at the study level using Egger’s regression test on the mean Fisher z per study.

### Meta-analysis

The primary effect size observed in this study was the Pearson correlation coefficient (*r*) between each Gustafson’s-derived variable and chronological age and analysed using the random-effects model. Fisher’s r-to-z transformation for each *r* was performed for variance stabilisation, and the pooled estimates were back-transformed to *r* for reporting. The three-level random-effects model assumes that the true effects are randomly sampled and approximately normally distributed at both the within-study (Level 2) and between-study (Level 3) levels; that effect sizes reported by the same study share a common study-level random effect, which accounts for their statistical dependency; and that the sampling variance of each effect size is known. Fisher’s z transformation renders the sampling distribution of each correlation approximately normal, with a variance that depends only on sample size (1/(*n* − 3)), thereby stabilising the variance across the range of r.

As the Gustafson-derived methodology normally employs multiple variables, one study can report multiple correlation coefficients and the effect sizes are statistically dependent. To accommodate this dependency, a three-level meta-analysis model was fitted. Where level 1 used the sampling variance of each effect size, Level 2 ($\:{{\upsigma\:}}_{w}^{2}$) calculates within-study variance among multiple effect sizes reported by the same study, and Level 3 ($\:{{\upsigma\:}}_{b}^{2}$) calculates the between-study variances. The corresponding heterogeneity statistics $\:{I}_{w}^{2}$ and $\:{I}_{b}^{2}$ quantify the proportion of total variance in within or between studies, and Cochran’s Q was used to test overall heterogeneity. All models were fitted using R version 4.5.2 (R Foundation for Statistical Computing, Vienna, Austria) with metafor statistical package with the rma.mv function^15^. Three subgroup analyses were conducted to observe the difference in the correlation coefficient between variables (i.e., attrition, periodontosis, etc.), sectioned or intact samples, and jaw position.

Regarding jaw analysis, studies that did not specify the origin of the jaw were excluded from the analysis; only studies that reported the tooth coming from the maxilla or mandible were considered in the meta-analysis. Studies with a high risk of bias were excluded from the meta-analysis a priori. Additionally, influence of any single study was assessed via leave-one-out estimation.

The certainty of evidence was assessed for each Gustafson-derived variable using the Grading of Recommendations Assessment, Development and Evaluation (GRADE) framework across five domains: risk of bias, inconsistency (between-study heterogeneity), indirectness, imprecision (width of the 95% confidence interval), and publication bias. Each variable was rated as high, moderate, low, or very low certainty^16^.

**Fig. 1:**
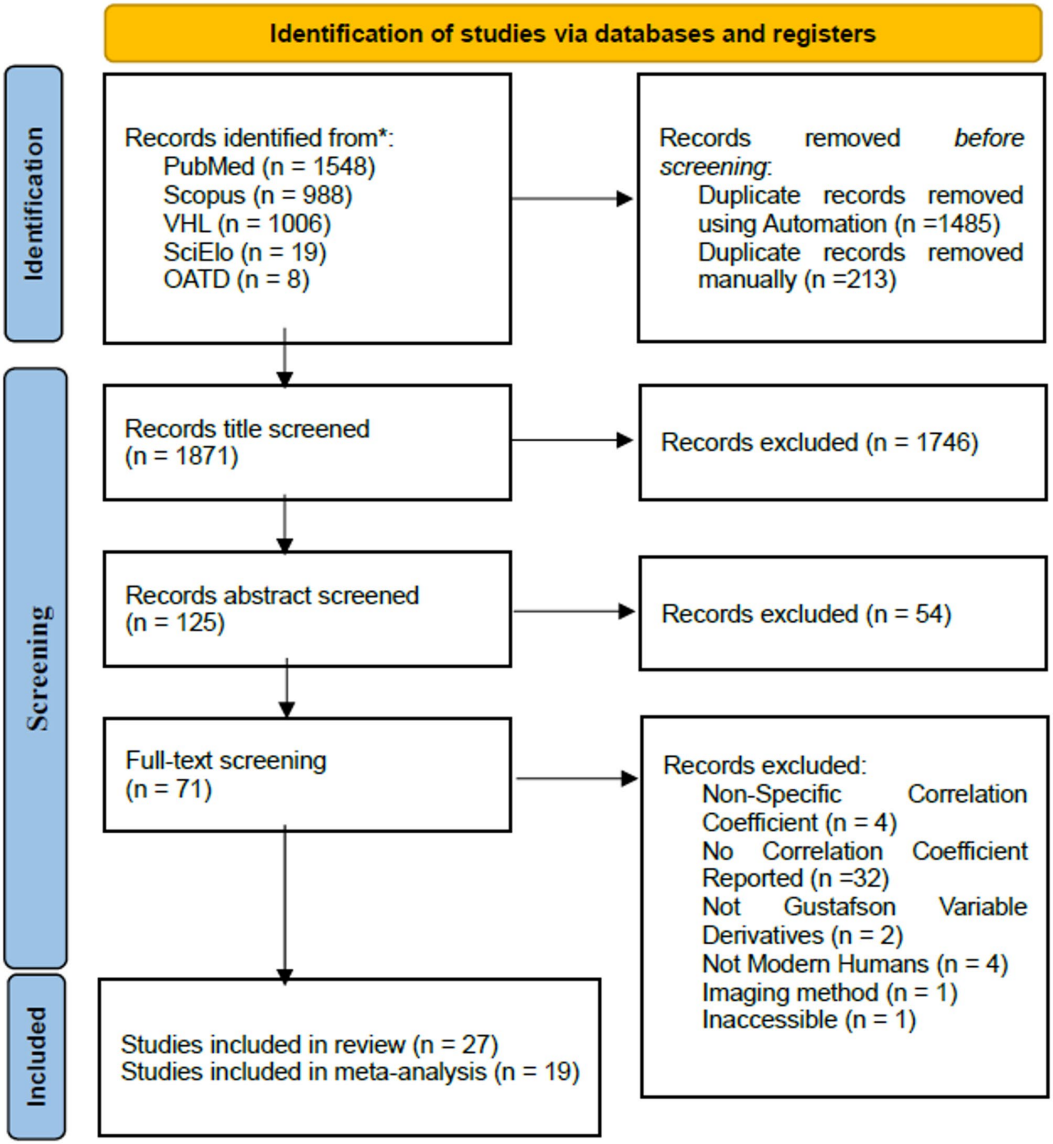
PRISMA flowchart for the systematic review.

## Results

The initial sample consisted of 3569 studies, leading to 27 eligible studies for the qualitative analysis, while 19 were eligible for the quantitative synthesis (Fig. 1). The reasons for exclusion in the full-text reading were the use of radiological imaging analysis (*n* = 1), lack of correlation value (*n* = 32), studies that have not addressed Gustafson’s variables derivatives (*n* = 2), unclear or not modern human populations (*n* = 4), studies reporting the overall combined correlation (*n* = 4), and inaccessibility (*n* = 1).

### Study characteristics

The 27 eligible studies ranged from 1990 to 2024, with a total of 151 reporting correlation coefficients (Table 2)^17, 18, 19, 20, 21, 22, 23, 24, 25, 26, 27, 28, 29, 30, 31, 32, 33, 34, 35, 36, 37, 38, 39, 40, 41, 42, 43^. From these 151 observations, the most commonly observed variables were Periodontosis (*n* = 46) and transparency of the root (*n* = 43), both of which were mostly calculated using the method proposed by Lamendin et al.^11^. Among the other variables, attrition (*n* = 9) was the least addressed.

**Table 2:** Studies included in the systematic review and meta-analysis.

Study ID	Author (Year)	Methodology	Observed variable						Sectioned or intact	*n*	Jaw	*r*-value range
			A	*P*	S	C	*R*	T				
1	Drusini et al. (1990)	Bang & Ramm						✓	Sectioned	33	Unspecified	0.49–0.55
2	Lopez-Nicolas et al. (1990)	Gustafson	✓	✓	✓	✓	✓	✓	Sectioned	173	Both	0.07–0.35
3	Solheim (1990)	Gustafson				✓			Sectioned	100	Both	0.07–0.4
		Johanson				✓						0.22–0.48
4	Drusini et al. (1991)	Length % of Translucent Dentin						✓	Intact	36	Unspecified	0.58–0.84
5	Solheim (1990)	Johanson			✓				Sectioned	78	Both	0.59–0.74
6	Solheim (1992)	Gustafson		✓					Sectioned	100	Both	0.01– 0.52
		Dalitz		✓								0.07–0.55
		Johanson		✓								0.07–0.5
7	Solheim & Kvaal (1993)	Gustafson					✓		Sectioned	78	Both	0.01–0.5
		Johanson					✓					-0.02–0.54
8	Thomas et al. (1994)	Length of Translucent Dentin						✓	Sectioned	55	Unspecified	0.59
		Length % of Translucent Dentin						✓				0.583
		Area of Translucent Dentin						✓				0.548
		Area % of Translucent Dentin						✓				0.467
9	Li & Ji (1995)	Average Stage of Attrition	✓						Intact	122–188	Both	0.94–0.97
10	Foti et al. (2001)	Lamendin		✓				✓	Intact	71	Both	-0.446–0.479
11	Acharya (2011)	Length of Translucent Dentin						✓	Sectioned	81	Unspecified	0.46–0.49
12	Acharya & Kumar (2011)	Johanson	✓	✓				✓	Intact	106	Unspecified	0.09–0.38
		Lamendin		✓				✓				0.05–0.4
		Average Stage of Attrition	✓									0.21
13	Kattappagari et al. (2014)	Area of Translucent Dentin						✓	Sectioned	50	Unspecified	0.77
		Length of Translucent Dentin						✓				0.14
14	Bommannavar & Kulkarni (2015)	Length of Translucent Dentin						✓	Sectioned	50	Unspecified	0.713–0.756
15	Shruthi et al. (2015	Length of Translucent Dentin						✓	Sectioned	150	Unspecified	0.97
16	Santoro et al. (2015)	Length of Translucent Dentin						✓	Intact	100	Unspecified	0.48
		Length % of Translucent Dentin						✓				0.53
17	Puneeth et al. (2016)	Length of Translucent Dentin						✓	Sectioned	120	Both	0.91
18	Ruiz et al. (2016)	Length of Translucent Dentin						✓	Intact	43	Unspecified	0.75
19	Shah et al. (2020)	Length of Translucent Dentin						✓	Sectioned	50	Both	0.93
20	Parra et al. (2020	Lamendin		✓				✓	Intact	693	Unspecified	0.77–0.9
21	Garizoain et al. (2020)	Lamendin		✓				✓	Intact	809	Unspecified	0.516–0.634
22	Limdiwala et al. (2021)	Lamendin		✓				✓	Intact	77	Unspecified	0.502–0.715
23	Baz et al. (2022)	Lamendin		✓				✓	Intact	74	Unspecified	0.237–0.362
24	Shylaja et al. (2022)	Length of Translucent Dentin						✓	Sectioned	150–300	Unspecified	0.973–0.975
25	Munoz et al. (2023)	Length of Translucent Dentin						✓	Intact	248	Mandible	0.775
		Length % of Translucent Dentin						✓				0.778
26	Kavya et al. (2023)	Lamendin		✓				✓	Intact	80	Both	0.646–0.693
		Average Stage of Attrition	✓									0.608
		Johanson	✓	✓				✓				0.648–0.83
27	Pinto et al. (2024)	Lamendin		✓				✓	Intact	141	Both	0.05–0.39

A = Attrition, P = Periodontosis, S = Secondary Dentin Deposition, C = Cementum Apposition, R = Root Resorption, T = Transparency of the Root, n = sample size.

Various adaptations of the Gustafson’s methodology have been reported, such as Johanson, Lamendin, and Dalitz. The methodology used by Bang & Ramm was listed as “Length of Translucent Dentin” to ensure homogeneity across the variables^44^. This adaptation included the ratio of dentin translucency length to root length (*n* = 7), average stage of attrition (*n* = 6), and area (*n* = 2) or the ratio of the area of dentin translucency to root area (*n* = 1). The most common observation was calculated from mandibular (*n* = 46) and maxillary teeth (*n* = 44), with some studies not differentiating between maxillary or mandibular teeth (*n* = 24) and some studies not specifying maxilla/mandible/both (*n* = 37).

### Risk of bias

Most studies had a moderate (*n* = 10), followed by low (*n* = 9), or high risk of bias (*n* = 8) according to the JBI Critical Appraisal Tool for Cross-Sectional Studies (Table 3)^14^. The most common bias (*n* = 20) observed in the included studies was subject details, where the studies should report the sample details such as tooth, jaw, or root count (i.e., maxillary first premolar, mandibular canine, single or multi-rooted teeth). Additionally, some studies did not address the validity of the measured outcome through inter- and/or intra-observer agreement such as Cohen’s kappa or Intraclass Correlation Coefficient (*n* = 19).

**Table 3:** Risk of bias assessment of included studies using JBI critical appraisal tools.

Study ID	Q1	Q2	Q3	Q4	Q5	Q6	Q7	Q8	Total	Category
1	x	x	x	✓	x	x	x	✓	25%	HIGH
2	✓	x	x	✓	x	x	x	✓	38%	HIGH
3	x	x	x	✓	✓	✓	x	✓	50%	MODERATE
4	✓	✓	✓	✓	x	x	x	✓	63%	MODERATE
5	x	x	x	✓	✓	✓	x	✓	50%	MODERATE
6	x	x	x	✓	✓	✓	x	✓	50%	MODERATE
7	x	x	✓	✓	✓	✓	✓	✓	75%	LOW
8	✓	x	x	✓	x	x	x	✓	38%	HIGH
9	✓	✓	x	✓	✓	✓	x	✓	75%	LOW
10	✓	x	✓	✓	✓	✓	x	✓	75%	LOW
11	✓	x	x	✓	x	x	x	✓	38%	HIGH
12	✓	✓	✓	✓	x	x	✓	✓	75%	LOW
13	✓	✓	x	✓	x	x	x	✓	50%	MODERATE
14	✓	x	x	✓	x	x	x	✓	38%	HIGH
15	✓	x	x	✓	x	x	x	✓	38%	HIGH
16	x	x	x	✓	✓	✓	x	✓	50%	MODERATE
17	✓	✓	✓	✓	x	x	✓	✓	75%	LOW
18	x	x	x	x	x	x	x	✓	13%	HIGH
19	✓	x	✓	✓	x	x	✓	✓	63%	MODERATE
20	x	x	✓	✓	✓	✓	x	✓	63%	MODERATE
21	x	x	x	✓	✓	✓	x	✓	50%	MODERATE
22	✓	✓	x	✓	x	x	x	✓	50%	MODERATE
23	✓	x	✓	✓	✓	✓	✓	✓	88%	LOW
24	✓	x	✓	✓	✓	✓	✓	✓	88%	LOW
25	✓	x	x	✓	x	x	x	✓	38%	HIGH
26	✓	✓	✓	✓	x	x	✓	✓	75%	LOW
27	✓	x	✓	✓	✓	✓	✓	✓	88%	LOW

### Meta-analysis

Nineteen studies with 131 data points were included in the meta-analysis after the exclusion of eight studies identified as having a high risk of bias (Study IDs: 1, 2, 8, 11, 14, 15, 18, and 25; Tables 2 and 3)^17, 24, 27, 30, 31, 34, 45, 46^. Table 4 reports the overall three-level meta-analysis results with their respective subgroup analyses.

**Table 4:** Three-level meta-analysis results for overall and subgroups correlation coefficients.

Groups	*n*(ES)	Weighted *r*	95% CI		Q(df)	$\:{\boldsymbol{I}}_{\boldsymbol{w}}^{2}$	$\:{\boldsymbol{I}}_{\boldsymbol{b}}^{2}$	GRADE Certainty Rating
			LB	UB				
Overall	19(131)	0.67	0.49	0.8	4388.13(130)	0.04	0.93	Low
Methodology								
Gustafson	3(30)	0.54	0.26	0.73	2024.25(123)	0.04	0.92	Low
Average Stage of Attrition	3(6)	0.53	0.19	0.76				
Lamendin	8(26)	0.53	0.29	0.72				
Dalitz^†^	1(10)	0.57	0.28	0.76				
Johanson	6(46)	0.57	0.30	0.75				
Area of Translucent Dentin^†^	1(1)	0.96	0.85	0.99				
Length of Translucent Dentin	5(7)	0.82	0.59	0.93				
Length % of Translucent Dentin	2(5)	0.80	0.63	0.89				
Variables*								
Attrition	3(8)	0.63	0.39	0.79	2719.97(125)	0.04	0.93	Low
Periodontosis	9(45)	0.60	0.37	0.76				
Secondary Dentin^†^	1(10)	0.65	-0.38	0.96				
Cementum Apposition†	1(20)	0.35	-0.67	0.91				
Root Resorption^†^	1(20)	0.28	-0.71	0.9				
Transparency of the Root	14(28)	0.75	0.59	0.86				
Tooth preparation								
Intact	11(44)	0.61	0.33	0.79	4172.85(129)	0.04	0.92	Low
Sectioned	8(87)	0.73	0.47	0.88				
Jaw								
Lower	6(44)	0.59	0.15	0.83	1703.42(86)	0.03	0.94	Low
Upper	6(44)	0.56	0.1	0.82				

n = Study number, ES = Effect size, r = correlation coefficient, CI = Confidence interval, LB = Lower bound, UB = Upper bound.

*Significant Difference between-category (*p* < 0.05).

^†^Pooled estimate derives from a single study and is reported for completeness only; interpretive caution required.

The overall weighted correlation between chronological age and Gustafson derived variables was 0.67 (95% CI 0.50–0.80) with low certainty due to variability in the methodology. Small heterogeneity was observed within the studies ($\:{I}_{w}^{2}$ = 0.04) and high heterogeneities between studies ($\:{I}_{b}^{2}$ = 0.93), indicating a homogenous observation within the study, but with significant difference between the observed variables reported between the studies, respectively.

Significant differences were also observed within the Gustafson’s derived variables, with the highest pooled correlation coefficient was found in the transparency of the root (*r* = 0.75, Low Certainty) and the lowest correlation coefficient was found for root resorption (*r* = 0.28, Low certainty). Further subgroup analysis indicated a higher correlation value between the sectioned and intact tooth correlation coefficients with low certainty, with the sectioned tooth possibly showing a higher correlation (*r* = 0.73) than the intact tooth (*r* = 0.61). In jaw position, the marginal pooled correlations were similar with no significant difference between the upper (*r* = 0.56) and lower jaws (*r* = 0.59) with low certainty.

### Sensitivity analysis

Three sensitivity analyses were performed. First, re-introducing the 8 high-risk-of-bias studies (i.e., returning to the full set of 151 effect sizes from all 27 studies) produced a pooled correlation of *r* = 0.68 (95% CI 0.54–0.78), within 0.01 of the primary estimates, and using risk of bias as a moderator in the three-level model was not statistically significant (*p* = 0.77). Second, leave-one-out re-estimation yielded pooled correlations ranging from *r* = 0.63 to *r* = 0.69, indicating that no single study disproportionately drove the overall result. Third, no significant asymmetry on Egger’s regression (z = 1.30, *p* = 0.21).

## Discussion

Although human dental identification is considered by the International Criminal Police Organisation as one of the primary identifiers alongside friction ridge analysis and DNA for human identification^47^, DAE itself is a secondary identifier, meaning that it can be useful in reconstructive dental profiling and enable a more straightforward comparisons using primary identifiers^48, 49^. In this systematic review, the direct observation DAE method for adults proposed by Gustafson was evaluated. The overall weighted *r* was 0.67, rated as low certainty using GRADE framework, mainly due to the diversity of methodology across the contributing studies. Although less precise than advanced adult DAE methodologies, including three-dimensional volumetric imaging^5^, direct visualization continues to offer practical value in forensic scenarios where rapid, low-cost age assessment is required and access to sophisticated technologies is constrained^50^.

Subgroup analyses suggested substantial variation among the six variables, with correlation coefficients ranging from a high of *r* = 0.75 for root transparency to a low of *r* = 0.28 for root resorption. When interpreting these subgroup estimates, a distinction must be drawn between the variables supported by multiple studies (attrition, periodontosis, and root transparency) and those derived from a single study each (secondary dentin deposition, cementum apposition, and root resorption); the latter are reported for completeness only, are flagged with the caution marker (†) in Table 4, and should not be ranked alongside, or interpreted as equivalent to, the better-supported variables. However, these results have the same underlying limitation as the primary weighted *r* that they are drawn from heterogeneous methodologies, despite evaluating the same variables. Furthermore, the estimates for secondary dentin deposition, cementum apposition, and root resorption were each derived from a single study. Even among the better-supported variables, the assumption that they carry equal weight or contribute equally to CA predictions should be avoided^51^. Consequently, when developing a new regression model based on Gustafson’s variables, each variable should be weighted according to its association with CA. Model evaluation techniques, such as Variable Importance Measures, can be employed to identify and remove less significant features in a regression model^52^.

The formation of translucent or sclerotic dentin is an intrinsic and progressive physiological process characterized by structural and physicochemical alterations within the root dentin. Several descriptions of this mechanism have been reported^36,53,54,55^. Generally, translucency becomes macroscopically observable from the third decade of life onward and is associated with the gradual accumulation of hydroxyapatite crystals within the dentinal tubules^36^. This process typically initiates in the apical region of the root and progressively extends coronally with age^53^. As mineral deposition increases, dentinal tubules become progressively occluded. In non-occluded dentin, the differences between the inter- and intra-tubular refractive indices cause light scattering, resulting in an opaque appearance^53^. Conversely, intra-tubular mineralization reduces these refractive differences, allowing greater light transmission through the tissue^54^. This optical continuity produces the characteristic translucent appearance observed in aged root dentin^55^. The higher pooled correlation coefficient observed for root translucency (*r* = 0.75) may be attributed to the relatively consistent and age-dependent nature of mineralization. Compared with other regressive variables, root translucency appears to be less susceptible to external pathological or environmental confounding influences, which may contribute to its stronger association with CA.

The methodological subgroup analysis showed that the method proposed by Lamendin et al. (1992)^11^ yielded the highest correlation coefficient. Lamendin’s method combines the calculation of periodontosis and the transparency of the root ratio relative to overall root height in an intact tooth, both of which demonstrated significant associations with age in the present analysis. Unlike root transparency, periodontosis has a multifactorial aetiology; its progression is heavily influenced by multiple confounding variables independent of chronological age (CA), such as periodontal disease, anatomical predispositions (e.g., tooth malposition and root prominence), and mechanical trauma^56^. Therefore, adapting a modified regression formula based on Lamendin’s method could assign greater weight to root transparency, or potentially eliminate the periodontal variable if found to be non-significant^26, 57^. Furthermore, since the conception of this study, several investigations focusing on transparency of the root have emerged with promising evidence on a global scale^36,58^. This evidence reinforces that the Low certainty of evidence observed in our analysis reflects the heterogeneity and indirectness among the included primary studies, rather than any inherent limitation of the methodology itself.

The sectioned teeth (*r* = 0.73) showed a numerically higher correlation with CA than intact teeth (*r* = 0.61), regardless of the methodology even without statistical significance. Sectioning a tooth provides a direct, unobstructed view of its internal regressive changes. This allows for more precise and repeatable measurements, thereby reducing measurement errors and strengthening the method performance. However, this increase in the correlation coefficient needs to be weighed against practical and ethical considerations, particularly in the context of DVI, where the laborious, multi-step process of extracting, sectioning, mounting, and grinding each tooth for analysis is often incompatible with the operational speed required for rapid identification^59^. While the scientific aspect of an identification process is important, normative (legal and ethical) or religious aspects must be respected by following jurisdictional protocols and applying the least invasive methods whenever possible.

The interpretation of these findings must consider limitations inherent to both the included evidence and the review process. Firstly, a substantial proportion of the included studies were judged to have a moderate or high risk of bias, particularly with respect to the description of study participants (Q2) and the validity and reliability of outcome measurements (Q7). Twenty studies provided insufficient information on participant characteristics, including the specific tooth type, jaw origin, and whether the teeth were sectioned. In addition, 19 studies did not evaluate measurement reliability through inter- and/or intra-observer agreement using statistics such as Cohen’s kappa or the Intraclass Correlation Coefficient.

Regarding the review process, subgroup analyses were limited by data scarcity, methodological variability, and indirectness. Three of the six Gustafson variables (i.e., secondary dentin deposition, cementum apposition, root resorption) were each represented by only a single study in the meta-analysis. Consequently, the corresponding estimates should be interpreted as evidence derived from individual studies rather than pooled effects. Given the limited evidence base and substantial between-study heterogeneity, these findings suggest that Adult DAE based on regressive changes—particularly those relying on direct examination—may exhibit considerable variability across studies and populations.

## Conclusion

When assessed through direct visualisation in adults, Gustafson-derived variables demonstrate a moderate overall association with chronological age (CA). However, pooled results require cautious interpretation due to the variability in individual markers and between-study heterogeneity. Root translucency is the most consistent predictor, whereas other regressive changes show weaker or more heterogeneous associations. Secondary dentin deposition, cementum apposition, and root resorption need further primary evidence to be assessed confidently and must be interpreted cautiously. Although sectioning increases measurement precision, methodological choices must balance analytical gain against ethical, legal, and operational considerations in forensic practice.

## Supplementary Information

Below is the link to the electronic supplementary material.

Supplementary Material 1

## Data Availability

The synthesised data that used in the current systematic review and meta-analysis are available from the first author upon reasonable request.
